# Association between neutrophil-lymphocyte ratio and all-cause and cardiovascular mortality in osteoarthritis patients from the NHANES 1999–2018 cohort

**DOI:** 10.1038/s41598-025-14465-3

**Published:** 2025-09-30

**Authors:** Jingyi Shen, Jiyuan Zhou, Feifei Xue, Wei Shen, Linyu Geng, Huayong Zhang

**Affiliations:** 1 Department of Rheumatology and Immunology, Nanjing Drum Tower Hospital Clinical College of Nanjing Medical University, Nanjing, Jiangsu China; 2 Department of Epidemiology, Harvard T. H. Chan School of Public Health, Boston, MA USA; 3 Department of Medicine, University of California San Francisco, San Francisco, CA USA; 4 Department of Rheumatology and Immunology, Nanjing Drum Tower Hospital, Affiliated Hospital of Medical School, Nanjing University, Nanjing, Jiangsu China

**Keywords:** Neutrophil-lymphocyte ratio, All-cause mortality, Cardiovascular mortality, Osteoarthritis, NHANES, Biomarkers, Prognostic markers, Rheumatology, Musculoskeletal system

## Abstract

This cross-sectional study aimed to investigate the correlation between the neutrophil-lymphocyte ratio (NLR) and all-cause mortality and cardiovascular mortality in osteoarthritis (OA) patients. We involved 3549 adults with OA from the National Health and Nutrition Examination Survey (NHANES) database (1999–2018). The optimal NLR threshold (2.53) was determined using maximally selected rank statistics. Kaplan-Meier (KM), weighted Cox regression, and restricted cubic spline (RCS) analyses were employed to assess the relationship between the NLR and mortality outcomes, with subgroup and sensitivity analyses evaluating the stability of the observed associations. Time-dependent receiver operating characteristic (ROC) curve analysis was conducted to evaluate the NLR prognostic accuracy for mortality across time points. During the 91-month median follow-up period, 843 patients died (256 from cardiovascular disease). Elevated NLR (≥ 2.53) was associated with increased risks of all-cause mortality (HR = 1.82) and cardiovascular mortality (HR = 2.50). Nonlinear correlations of the NLR with mortality outcomes were observed. ROC analysis demonstrated superior NLR predictive capability for all-cause and cardiovascular mortality compared to individual blood cell types. Elevated NLR is independently associated with increased risks of all-cause mortality and cardiovascular mortality in OA patients.

**Supplementary Information:**

The online version contains supplementary material available at 10.1038/s41598-025-14465-3.

## Introduction

Osteoarthritis (OA) is a chronic joint disorder affecting the articular cartilage, subchondral bone, and adjacent soft tissues^1^. The global burden of OA continues to rise, with disability-adjusted life-years reaching 36.6 million by 2050^2,3^. Numerous studies have demonstrated that OA increases the risk of cardiovascular disease^4–9^. Furthermore, OA patients exhibit significantly higher all-cause mortality and cardiovascular mortality rates compared to the general population^10–13^. OA develops due to a combination of local and systemic factors, including mechanical stress, aging, obesity, and genetics^14^. These factors trigger joint damage through localized and systemic inflammation, increasing levels of cytokines, adipokines, reactive oxygen species, and immune cells, which in turn exacerbate inflammation and autoimmune responses^15^. Chronic low-grade inflammation and immune activation constitute a shared pathological basis between OA and cardiovascular disease^16^. A recent study has further demonstrated that shared genetic factors may partially explain the association between OA and cardiovascular disease^17^. Furthermore, the mechanistic link between OA and mortality remains unclear. Current evidence suggests that the impact of OA on mortality may be mediated by depression, functional impairment, reduced quality of life, and the use of non-steroidal anti-inflammatory drugs^18–21^. Consequently, identifying practical prognostic biomarkers is imperative for preventing OA progression and reducing associated mortality.

Inflammation is pivotal in OA pathogenesis, exerting a central influence throughout the entire disease course, from initiation to progression^22^. The neutrophil-lymphocyte ratio (NLR), a readily available and economical inflammatory indicator, has garnered attention for its prognostic value across multiple diseases^23–28^. A study using National Health and Nutrition Examination Survey (NHANES) data demonstrated a significant correlation of the NLR with all-cause mortality and specific mortality from specific causes, including cancer, lower respiratory diseases, cerebrovascular disease, and heart disease^29^. Furthermore, NLR was found to be associated with both all-cause mortality and cardiovascular mortality in populations with hypertension, diabetes mellitus, chronic obstructive pulmonary disease, rheumatoid arthritis, and coronary heart disease^30–34^. These results highlight the potential of the NLR as a robust biomarker for predicting disease outcomes and overall mortality risk. In OA research, the existing literature has reported significantly higher NLR values in OA patients compared with healthy controls^35^. Two studies identified an NLR ≥ 2.1 as a predictive factor for severe knee OA^36,37^. However, a study suggested that NLR does not have high diagnostic or prognostic value in determining the severity of knee OA^38^. Notably, current research has primarily focused on the association between NLR and OA progression, whereas investigations into NLR’s potential role in mortality among OA patients remain scarce.

Consequently, we conducted this cross-sectional study to clarify the correlation between the NLR and all-cause and cardiovascular mortality in OA patients via updated data from the NHANES database (1999–2018). By identifying an optimal NLR threshold and examining its predictive accuracy, we aim to gain a deep understanding of the prognostic utility of the NLR in OA management.

## Material and methods

### Study population

**Fig. 1:**
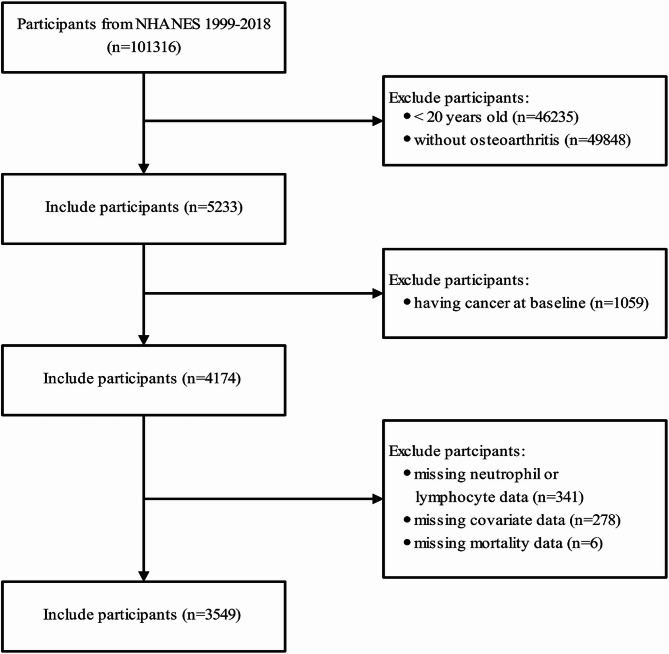
Flow diagram of the patients.

This study employed a cross-sectional design to investigate the associations between the NLR and all-cause and cardiovascular mortality in OA patients. Data from ten cycles of the NHANES survey (1999–2000, 2001–2002, 2003–2004, 2005–2006, 2007–2008, 2009–2010, 2011–2012, 2013–2014, 2015–2016, and 2017–2018) comprising a total of 101,316 patients, were reviewed. Patients diagnosed with OA were selected to evaluate the correlations of the NLR with mortality risk. Initially, 5233 patients with OA aged ≥ 20 years were enrolled. After excluding individuals with cancer diagnoses at baseline (*n* = 1059), those with missing NLR data (*n* = 341), those lacking the required covariate information (*n* = 278), and those without all-cause mortality data (*n* = 6), the final analysis included 3549 participants. (Fig. 1).

### Definition of variables

OA was ascertained through self-reported physician diagnoses in the NHANES questionnaire. Those who reported OA participated.

The NLR was calculated via complete blood count data measured by automated hematology analyzers at the NHANES mobile examination center. It is the ratio of neutrophils to lymphocytes.

### Determination of mortality

Mortality status was ascertained from the NHANES public-use linked mortality file, which was matched with the National Death Index through a probabilistic match. Cardiovascular mortality was categorized as I00–I09, I11, I13, and I20–I51 on the basis of the 10th Revision of the International Classification of Diseases^39^. All-cause mortality was any cause of death. The interval between the initial interview and the death or the termination of the follow-up determined the duration of the follow-up.

### Covariates

This analysis accounted for various demographic characteristics, lifestyle factors, and clinical information as covariates, including age, sex (male, female), race (Mexican American, other Hispanic, non-Hispanic White, non-Hispanic Black, other/multiracial), education level (less than high school, high school or equivalent, college or above), smoking status (never smoker, former smoker, current smoker), drinking status (nondrinker, 1 to < 5 drinks/month, 5 to < 10 drinks/month, ≥ 10 drinks/month), body mass index (BMI), diabetes (yes, no), and hypertension (yes, no). Diabetes was defined as a self-reported doctor’s diagnosis, use of anti-diabetic medication or insulin, a hemoglobin A1c level of ≥ 6.5%, or a fasting glucose level of ≥ 126 mg/dL. Hypertension was defined as a self-reported doctor’s diagnosis, use of antihypertensive medication at baseline, an average systolic blood pressure of ≥ 140 mmHg, or an average diastolic blood pressure of ≥ 90 mmHg. These covariates, which could confound or modify the results, were identified on the basis of prior studies and expert clinical evaluations^40,41^.

### Statistical analysis

Consistent with the Centers for Disease Control and Prevention guidelines, the complex survey design factors for the NHANES were accounted for in all analyses. Normally distributed continuous variables are expressed as the means (standard deviations), whereas nonnormally distributed continuous variables are expressed as the medians (interquartile ranges). Categorical variables are displayed as unweighted counts (weighted percentage, %). Student’s t-test was employed to evaluate normally distributed continuous variables, whereas the Mann-Whitney U test was applied for nonnormally distributed continuous variables. For categorical variables, the Rao-Scott adjusted chi-square test was employed.

The maximally selected rank statistics method was used to determine the optimal threshold for the NLR^15^. Kaplan-Meier (KM) analysis was used to evaluate survival in different NLR groups. Two weighted Cox regression models were used to examine the associations of the NLR with all-cause mortality and cardiovascular mortality: Model 1 was adjusted for age and sex data; Model 2 was adjusted for age, sex, race, education level, smoking status, drinking status, BMI, diabetes, and hypertension data. To illustrate the nonlinear dose-response relationships between the NLR and mortality outcomes, restricted cubic spline (RCS) analysis with three knots was employed.

Subgroup analysis was conducted by age, sex, race, smoking status, drinking status, BMI, diabetes, and hypertension. We conducted a sensitivity analysis by excluding patients who died in the first two years and those with diabetes, thereby minimizing the potential for reverse causality to evaluate the robustness of the associations. Additionally, we employed time-dependent receiver operating characteristic (ROC) curve analysis to examine the accuracy of the NLR in predicting survival outcomes at various time points.

Data analyses were conducted with R version 4.3.2 with the ‘maxstat,’ ‘gtsummary,’ ‘dplyr,’ ‘survey,’ ‘survival,’ ‘survminer,’ ‘ggplot2,’ ‘rms,’ and ‘time ROC’ packages. Two-sided P values less than 0.05 were considered significant.

## Results

### Participant characteristics

A total of 3549 individuals with OA were identified from the NHANES 1999–2018 cycles, with a median age of 61.00 years and a female predominance (65.45%). The NLR threshold for survival was 2.53; participants were divided into a high NLR group (NLR ≥ 2.53, *n* = 1109) and a low NLR group (NLR < 2.53, *n* = 2440) (Supplementary Fig. S1). Participants with high NLRs tended to be older and more likely to be non-Hispanic White. While females comprised the majority in both groups, the high NLR group demonstrated a higher proportion of males relative to the low NLR group. Furthermore, participants with elevated NLR exhibited a higher incidence of diabetes and elevated neutrophil and monocyte counts, along with reduced lymphocyte counts (Table 1).

**Table 1:** Baseline characteristics of osteoarthritis patients.

Characteristic	Overall *N* = 3549	Low NLR (< 2.53) *N* = 2440	High NLR (≥ 2.53) *N* = 1109	*P* value
Age (years)	61.00 (52.00,70.00)	60.00 (52.00,69.00)	62.00 (52.00,72.00)	0.015
Sex, n (%)				0.003
Male	1263 (34.55)	812 (32.47)	451 (39.05)	
Female	2286 (65.45)	1628 (67.53)	658 (60.95)	
Race, n (%)				< 0.001
Mexican American	332 (3.12)	221 (2.93)	111 (3.52)	
Other Hispanic	238 (2.80)	176 (2.99)	62 (2.39)	
Non-Hispanic White	2171 (81.94)	1389 (79.57)	782 (87.06)	
Non-Hispanic Black	571 (7.00)	471 (8.54)	100 (3.66)	
Other/multiracial	237 (5.14)	183 (5.96)	54 (3.37)	
Education level, n (%)				0.053
Less than high school	783 (13.90)	538 (12.88)	245 (16.10)	
High school or equivalent	860 (24.73)	602 (25.93)	258 (22.13)	
College or above	1906 (61.37)	1300 (61.19)	606 (61.77)	
Smoking status, n (%)				0.621
Never smoker	1704 (48.32)	1200 (49.06)	504 (46.71)	
Former smoker	1228 (35.17)	825 (34.76)	403 (36.05)	
Current smoker	617 (16.52)	415 (16.18)	202 (17.24)	
Drinking status, n (%)				0.065
Nondrinker	1283 (31.64)	907 (32.89)	376 (28.94)	
1–5 drinks/month	1585 (46.19)	1077 (44.62)	508 (49.58)	
5–10 drinks/month	179 (5.96)	128 (6.57)	51 (4.63)	
≥ 10 drinks/month	502 (16.21)	328 (15.91)	174 (16.84)	
BMI (kg/m^2^)	29.60 (25.56,34.20)	29.63 (25.62,33.88)	29.50 (25.41,35.48)	0.260
Diabetes, n (%)	971 (22.80)	647 (20.98)	324 (26.73)	0.005
Hypertension, n (%)	2332 (60.41)	1585 (59.95)	747 (61.43)	0.586
Neutrophil, ×10^9^ /L	4.10 (3.30,5.20)	3.70 (3.00,4.50)	5.20 (4.30,6.50)	< 0.001
Lymphocyte, ×10^9^ /L	2.00 (1.60,2.40)	2.10 (1.80,2.60)	1.60 (1.30,1.90)	< 0.001
Platelet, ×10^9^ /L	240.00 (202.00,287.00)	239.00 (202.00,284.00)	244.00 (200.00,291.00)	0.407
Monocyte, ×10^9^ /L	0.50 (0.40,0.70)	0.50 (0.40,0.70)	0.60 (0.50,0.70)	< 0.001

Data are displayed as median (interquartile range) or unweighted frequency counts (weighted percentages). NLR: neutrophil-lymphocyte ratio; BMI: body mass index.

### Associations between the NLR and all-cause and cardiovascular mortality

**Fig. 2:**
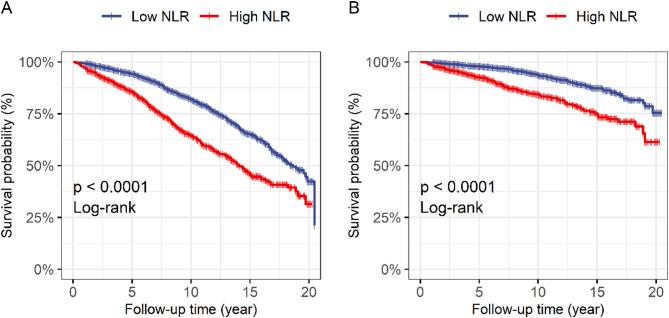
Kaplan-Meier analysis of the relationship between the neutrophil-lymphocyte ratio and mortality in osteoarthritis patients. (A) All-cause mortality; (B) Cardiovascular mortality.

Following a 91-month median follow-up period, 843 patients died, including 256 from cardiovascular disease. For the all-cause mortality analysis, 3549 patients were included. In the cardiovascular mortality analysis, of the 843 deceased patients, 587 died from causes other than cardiovascular disease. Therefore, these 587 patients were excluded from the cardiovascular mortality analysis, leaving 2962 patients for this specific analysis. The KM analysis revealed significantly lower overall survival and cardiovascular survival probabilities in patients with high NLRs than in those with low NLRs (Fig. 2). Two Cox regression models were used to assess the correlation of the NLR with mortality. After multivariate adjustment for age, sex, race, education level, smoking status, drinking status, BMI, diabetes, and hypertension (Model 2), patients in the high NLR category had significantly increased all-cause mortality (hazard ratio (HR) 1.82, 95% confidence interval (CI) 1.51–2.20, *P* < 0.001) and cardiovascular mortality (HR 2.50, 95% CI 1.72–3.64, *P* < 0.001) risk compared with those in the low NLR category. Moreover, the NLR was positively correlated with all-cause (Model 2, HR 1.15, 95% CI 1.09–1.22, *P* < 0.001) and cardiovascular mortality (Model 2, HR 1.23, 95% CI 1.14–1.32, *P* < 0.001) (Table 2).

**Table 2:** Weighted Cox regression analysis of the relationship between the neutrophil-lymphocyte ratio and mortality in osteoarthritis patients.

	NLR levels			Per 1 increment	*P* value
	Low NLR (< 2.53)	High NLR (≥ 2.53)	*P* value		
All-cause mortality					
Model 1	1 (ref)	1.85 (1.53, 2.24)	< 0.001	1.15 (1.09, 1.22)	< 0.001
Model 2	1 (ref)	1.82 (1.51, 2.20)	< 0.001	1.15 (1.09, 1.22)	< 0.001
Cardiovascular mortality					
Model 1	1 (ref)	2.56 (1.75, 3.74)	< 0.001	1.20 (1.11, 1.29)	< 0.001
Model 2	1 (ref)	2.50 (1.72, 3.64)	< 0.001	1.23 (1.14, 1.32)	< 0.001

Model 1: adjusted for age and sex. Model 2: adjusted for age, sex, race, education level, smoking status, drinking status, BMI, diabetes, and hypertension data. NLR: neutrophil-lymphocyte ratio; BMI: body mass index.

**Fig. 3:**
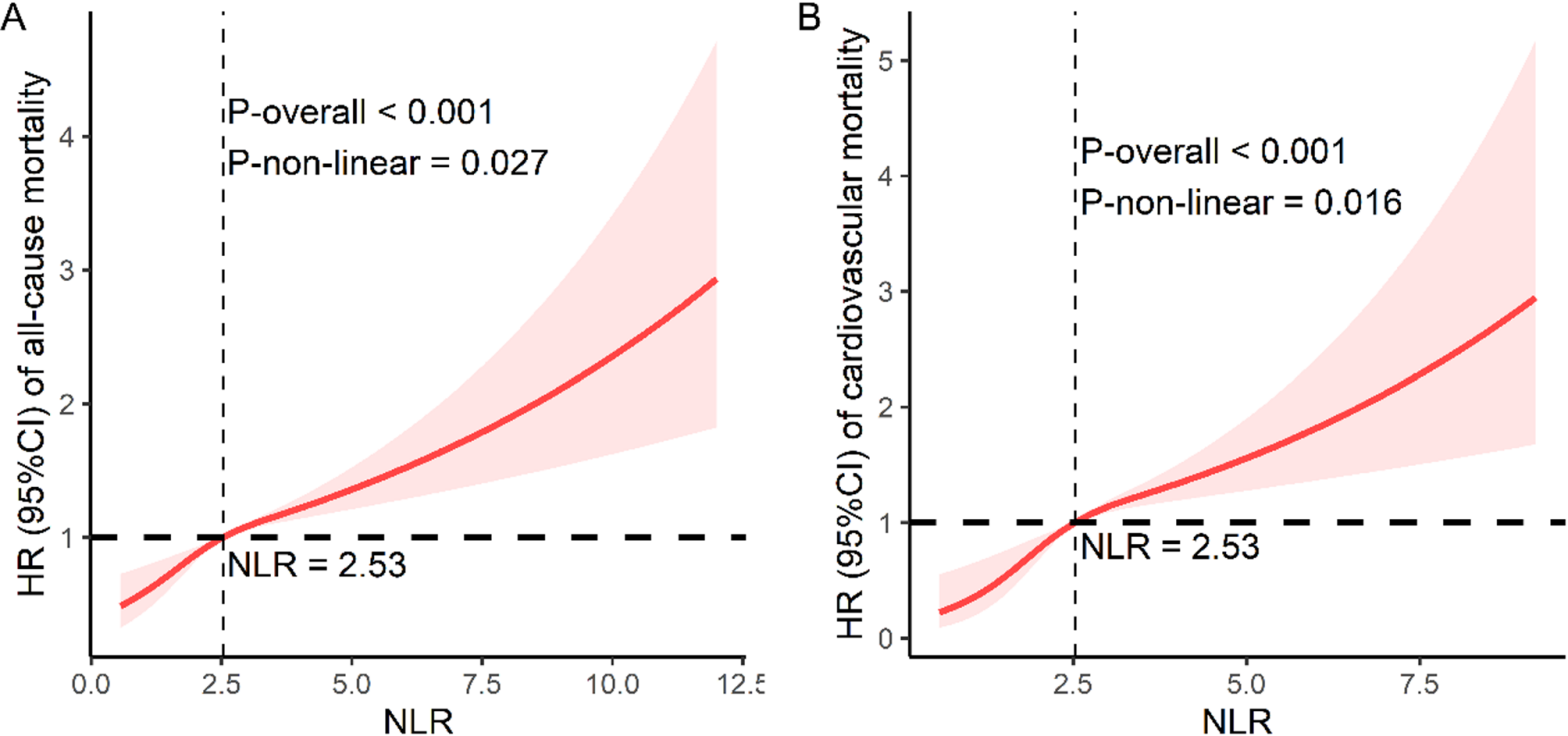
Restricted cubic spline analysis between the neutrophil-lymphocyte ratio and mortality in osteoarthritis patients. NLR breakpoint: 2.53. Adjusted for age, sex, race, education level, smoking status, drinking status, BMI, diabetes, and hypertension data. (A) All-cause mortality; (B) Cardiovascular mortality. BMI: body mass index.

The multivariate adjustment (Model 2) RCS plots revealed a nonlinear relationship of the NLR with all-cause mortality and cardiovascular mortality (P for nonlinearity = 0.027 and = 0.016, respectively) (Fig. 3).

### Subgroup and sensitivity analyses

**Fig. 4:**
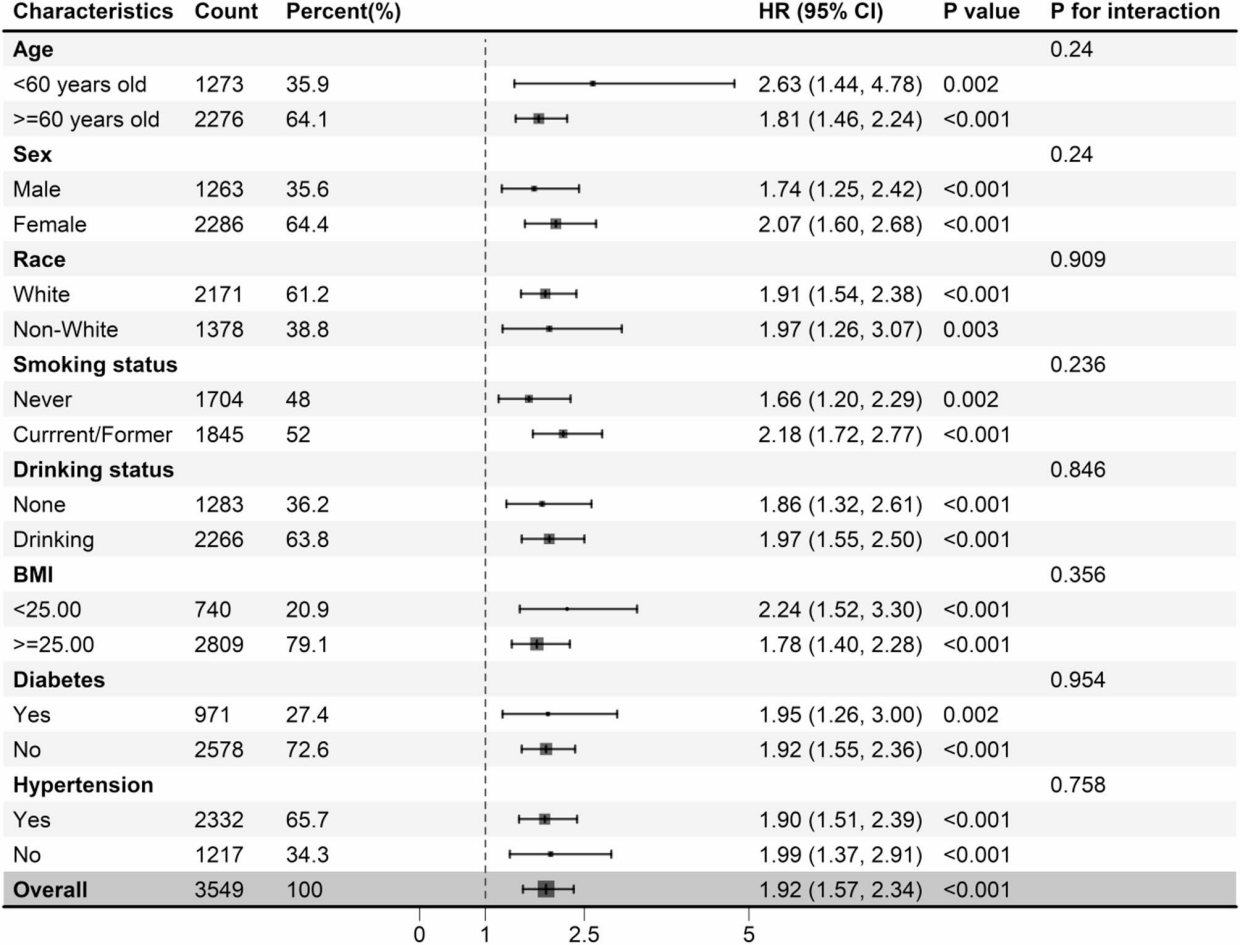
Subgroup analysis of the relationship between the neutrophil-lymphocyte ratio and all-cause mortality in osteoarthritis patients. Adjusted for age, sex, race, education level, smoking status, drinking status, BMI, diabetes, and hypertension data, with the exception of stratifying factors. BMI: body mass index.

**Fig. 5:**
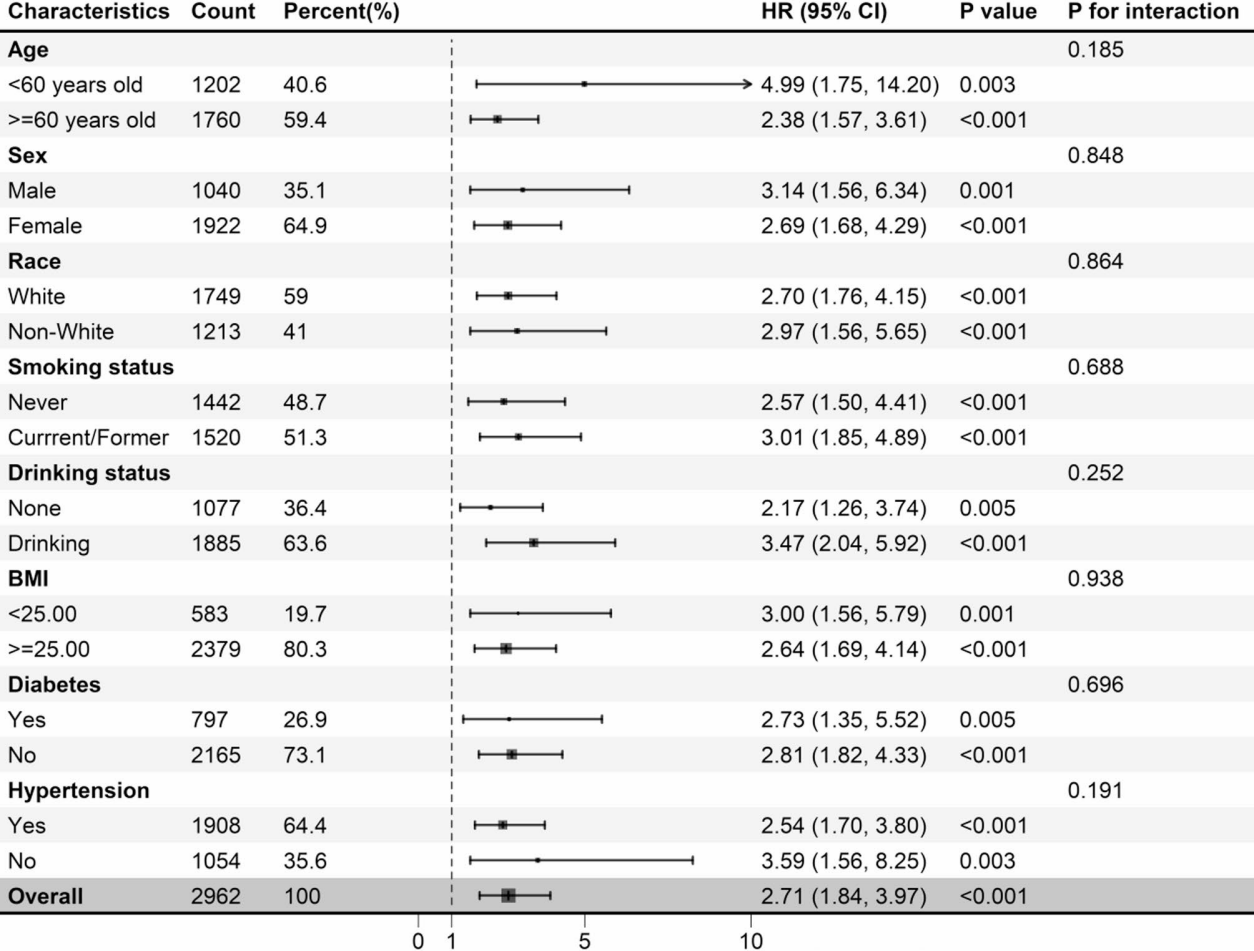
Subgroup analysis of the relationship between the neutrophil-lymphocyte ratio and cardiovascular mortality in osteoarthritis patients. Adjusted for age, sex, race, education level, smoking status, drinking status, BMI, diabetes, and hypertension data, with the exception of stratifying factors. BMI: body mass index.

The superiority of high NLRs over low NLRs in predicting survival in individuals with OA was consistently observed across various subgroups stratified by age (< 60, ≥ 60 years), sex (male, female), race (white, non-white), smoking status (never, current/former smoker), drinking status (none, drinking), BMI (< 25, ≥ 25 kg/m^2^), diabetes (yes, no), and hypertension (yes, no). There was no significant interaction between the NLR groups and stratification variables (Figs. 4 and 5). After excluding patients who died within the first two years, consistent links between the NLR and mortality outcomes remained observable (Supplementary Table S1). Similarly, after participants with diabetes were excluded, the NLR was significantly associated with mortality outcomes (Supplementary Table S2).

### ROC analysis

**Fig. 6:**
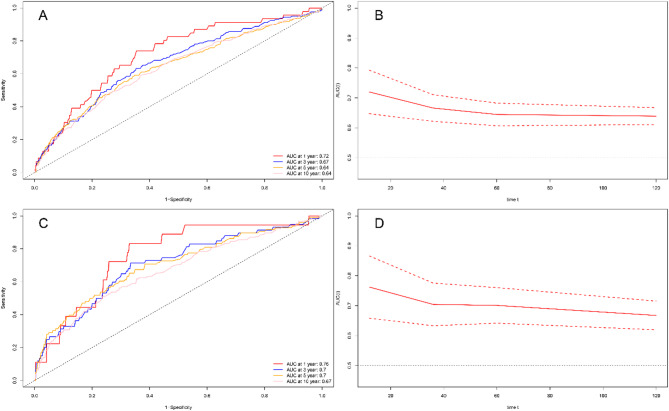
Time-dependent receiver operating characteristic curve analysis of the neutrophil-lymphocyte ratio for mortality prediction in osteoarthritis patients. (**A**,**B**) All-cause mortality; (**C**,**D**) Cardiovascular mortality.

Time-dependent ROC analysis revealed that the area under the curve (AUC) values of the NLR were 0.72, 0.67, 0.64, and 0.64 for predicting all-cause mortality at 1, 3, 5, and 10 years. The AUC values for the NLR were 0.76, 0.70, 0.70, and 0.67 for predicting cardiovascular mortality at 1, 3, 5, and 10 years (Fig. 6). We also assessed the ability of individual blood cell types, including lymphocytes, monocytes, neutrophils, and platelets, to predict all-cause and cardiovascular mortality. The findings indicated that the NLR was more predictive of mortality outcomes than other individual blood cells (Supplementary Fig. S2-S5).

## Discussion

This study represents one of the largest and most comprehensive investigations into the correlation between the NLR and all-cause and cardiovascular mortality in OA patients. We provide strong evidence that high NLRs are independently correlated with increased all-cause and cardiovascular mortality risks in OA patients. The findings of a nonlinear link between the NLR and mortality outcomes, along with the consistency of these findings across various subgroups and sensitivity analyses, support the potential of the NLR as a prognostic biomarker in patients with OA.

NLR is a cost-effective, easily measured, and accessible biomarker essential for clinical diagnosis, disease monitoring, and prognosis in various conditions. The NLR has recently been recognized as a valuable prognostic marker across a range of diseases, including cancer, diabetes, hypertension, and cardiovascular disease^30,31,42,43^. Findings from a prospective cohort study demonstrated that high NLR levels were strongly correlated with increased mortality in centenarian^44^. NLR demonstrates significant potential as a reliable biomarker for predicting disease prognosis, as highlighted by these findings.

Identifying an optimal NLR threshold of 2.53 is a significant contribution. The typical adult NLR ranges between 1 and 2, with values between 2.3 and 3.0, suggesting the presence of pathological conditions^45^. Previous studies with smaller sample sizes proposed an NLR threshold of 2.1 for predicting severe knee OA^36,37^. However, our more extensive scale study, which accounts for mortality risk factors, indicates that 2.53 may better reflect OA pathological features and clinical outcomes. The superiority of the NLR over individual cell counts in predicting mortality, as previously reported in studies on NLR and mortality in individuals with hypertension^30^was also confirmed in our analysis. Specifically, we found that NLR outperformed individual counts of neutrophils, lymphocytes, monocytes, and platelets in mortality prediction, highlighting its value as an integrated marker of systemic inflammation.

Previous studies have primarily focused on the predictive value of the NLR for OA onset, leaving its prognostic role within individuals with OA largely unexplored. A study based on the UK Biobank confirmed the correlation between increased NLR and OA risk, yet subgroup analysis indicated no significant link in males or individuals under 60 years of age^46^. Our study, in contrast, verified the robustness of the consistent relationship between the NLR and mortality outcomes while also indicating the broad prediction applicability of the NLR across diverse demographic characteristics, lifestyles, and comorbidity subgroups. The differing findings regarding demographic-specific associations could be attributed to differences in research objectives, outcome measures, and sample characteristics. Our focus on mortality outcomes may provide more specific insight into the prognostic value of the NLR, particularly in severe OA progression. Notably, while some studies suggest that the NLR is a key predictor of severe knee OA, the UK Biobank study did not find a precise correlation between the NLR and knee OA^36,37,46^. Due to the lack of specific OA-type data in the NHANES database, we were unable to explore this relationship further. Future clinical cohort studies are essential for clarifying the connections between the NLR, specific types of OA, and their prognostic implications.

In comparative analyses with other inflammatory markers, the NLR demonstrated superior predictive capability. In a recent study focusing on patients with diabetes mellitus and prediabetes, NLR and the systemic immune-inflammation index (SII) were identified as predictors of cardiovascular risk and mortality, with NLR exhibiting significantly better predictive performance than SII^47^. Yao et al. evaluated the predictive capacity of NLR, platelet-to-lymphocyte ratio (PLR), and C-reactive protein (CRP) for in-hospital mortality in patients with acute exacerbation of chronic obstructive pulmonary disease, finding that NLR outperformed PLR and CRP, with sensitivity and specificity of 81.08% and 69.17%, respectively^48^. Another group of researchers assessed the prognostic performance of the systemic inflammatory response index (SIRI), NLR, PLR, monocyte-to-lymphocyte ratio (MLR), and glucose-to-lymphocyte ratio (GLR) for successful mechanical ventilation weaning and 30-day mortality in intensive care unit patients with traumatic brain injury, finding that NLR showed good predictive accuracy for 30-day mortality with an AUC of 0.752^49^. NLR has consistently demonstrated robust prognostic utility across multiple disease conditions, while no studies have directly compared NLR with other inflammatory markers in predicting mortality in patients with OA. Therefore, future research could explore the interrelationships among these biomarkers to identify more reliable prognostic indicators for OA.

The association of the NLR with increased mortality in OA potentially stems from inflammation and the immune response. The subchondral bone, articular cartilage, and adjacent soft tissues in OA joints are major sites of inflammation, with numerous mediators and receptors driving pain and tissue damage^22^. In addition to local inflammation, low-grade inflammatory states related to aging, obesity, type 2 diabetes, and metabolic syndrome also significantly contribute to OA progression^14,50^. Macrophages, neutrophils, and various inflammatory cytokines collaborate to regulate joint inflammation^51^. Neutrophil elastase, which is released by neutrophils during degranulation, contributes to cartilage collagen destruction by directly activating latent metalloproteinase-13 in OA^52^. Wang et al. revealed that neutrophil elastase can induce chondrocyte apoptosis via the caspase-3 signaling pathway, further accelerating OA progression^53^. T cells are also significant immune cells within OA synovial fluid and synovial tissue^51^and an imbalance in Th1/Th2 and Th17/Treg cells is crucial to OA progression^54^. In OA, the frequencies of CD4^+^ T cells and B cells were lower than those in healthy controls, reflecting the immune dysfunction associated with OA^55^. All findings mentioned suggest that neutrophils and lymphocytes, key components of the NLR, are crucial to the progression of OA.

This study is subject to several limitations. First, despite the large sample size and extended follow-up, causality cannot be established in this cohort study. Second, the NHANES provides complete blood count data at baseline, and we could not obtain data on these levels during the follow-up period. Third, while we accounted for several potential confounders in our multivariable models, unmeasured factors may still influence the NLR. Moreover, we conducted a complete case analysis to address missing data by excluding individuals with missing values for the independent variables, covariates, and dependent variables. While this approach allows the analysis of available data, it may introduce selection bias as individuals with missing data are excluded, thus limiting the external validity of our findings. Furthermore, this study was based on the American population, which might not be generalizable to other populations. Thus, additional multicenter randomized clinical trials and prospective studies are needed to confirm the prognostic significance of the NLR in patients with OA.

## Conclusion

In conclusion, our research demonstrated that elevated NLR is independently related to increased all-cause and cardiovascular mortality risks in individuals with OA. The NLR could serve as a practical inflammatory marker for risk assessment in this population.

## Supplementary Information

Below is the link to the electronic supplementary material.

Supplementary Material 1

## Data Availability

All the data analyzed during this study are available on the NHANES website (https://wwwn.cdc.gov/nchs/nhanes/Default.aspx).

## Abbreviations

OA: Osteoarthritis
NLR: Neutrophil-lymphocyte ratio
NHANES: National Health and Nutrition Examination Survey
BMI: Body mass index
KM: Kaplan-Meier
RCS: Restricted cubic spline
ROC: Receiver operating characteristic
HR: Hazard ratio
CI: Confidence interval
AUC: Area under the curve
SII: Systemic immune-inflammation index
PLR: platelet-to-lymphocyte ratio
CRP: C-reactive protein
SIRI: Systemic inflammatory response index
MLR: Monocyte-to-lymphocyte ratio
GLR: Glucose-to-lymphocyte ratio
