# Supplementary material for: Association between neutrophil-lymphocyte ratio and all-cause and cardiovascular mortality in osteoarthritis patients from the NHANES 1999–2018 cohort
Source: Sci Rep. 2025 Sep 30;15:34061. doi: 10.1038/s41598-025-14465-3 (PMC12484823; doi:10.1038/s41598-025-14465-3)
Supplement: Supplementary file 1 — Supplementary Material 1 [file 41598_2025_14465_MOESM1_ESM.pdf]

Supplementary Figures

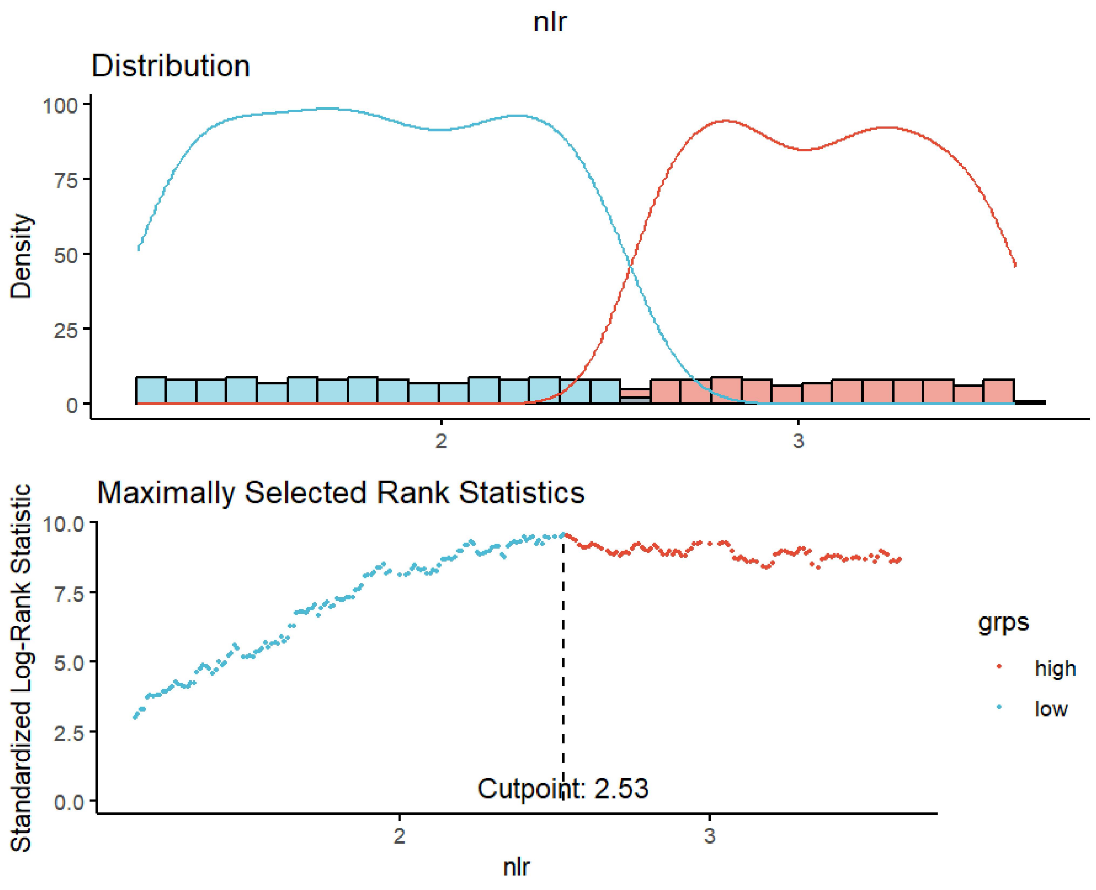

Supplementary Figure S1. Maximally selected rank statistics for the optimal neutrophil-lymphocyte ratio threshold determination.

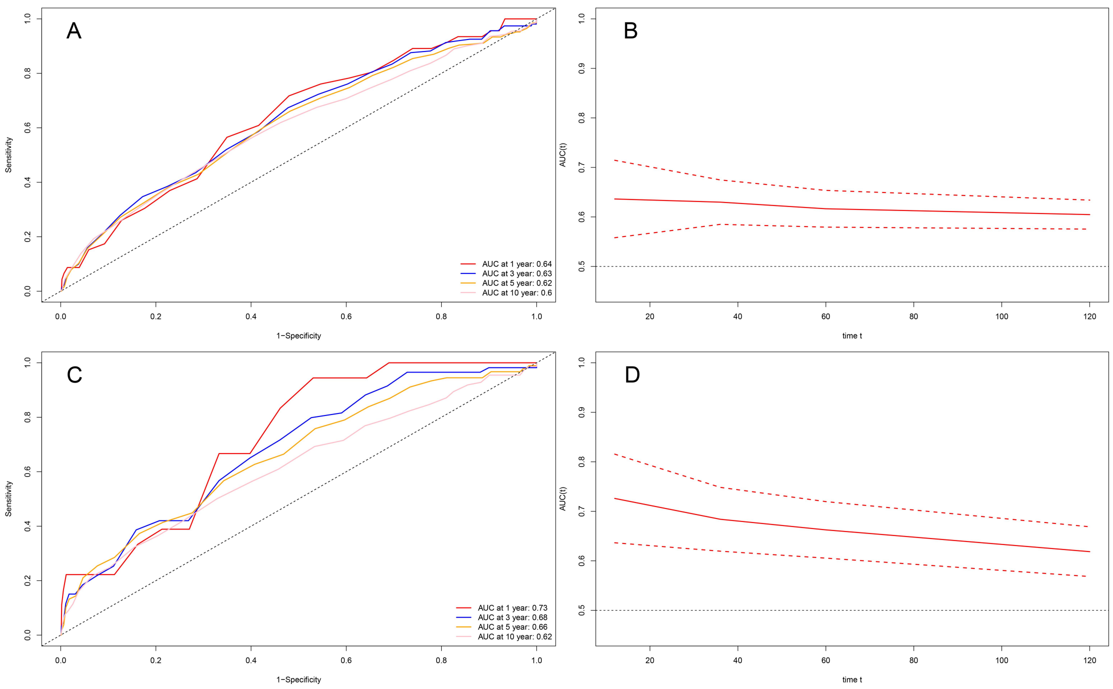

Supplementary Figure S2. Time-dependent receiver operating characteristic curve analysis of lymphocytes for mortality prediction in osteoarthritis patients. (A, B) All-cause mortality; (C, D) Cardiovascular mortality.

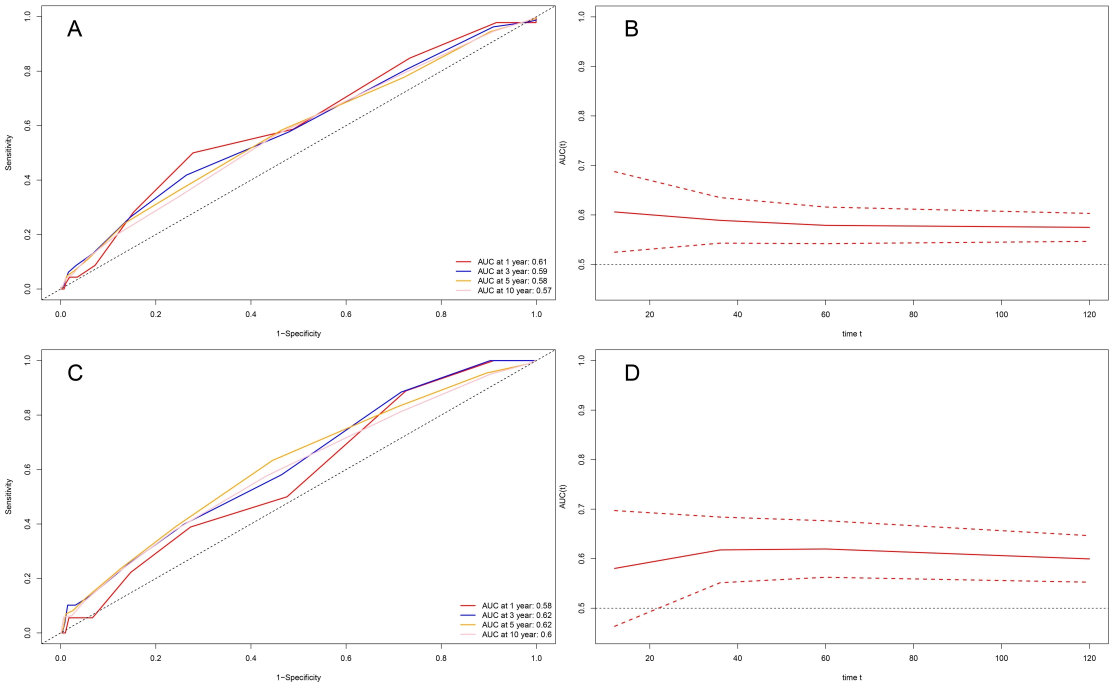

Supplementary Figure S3. Time-dependent receiver operating characteristic curve analysis of monocytes for mortality prediction in osteoarthritis patients. (A, B) All-cause mortality; (C, D) Cardiovascular mortality.

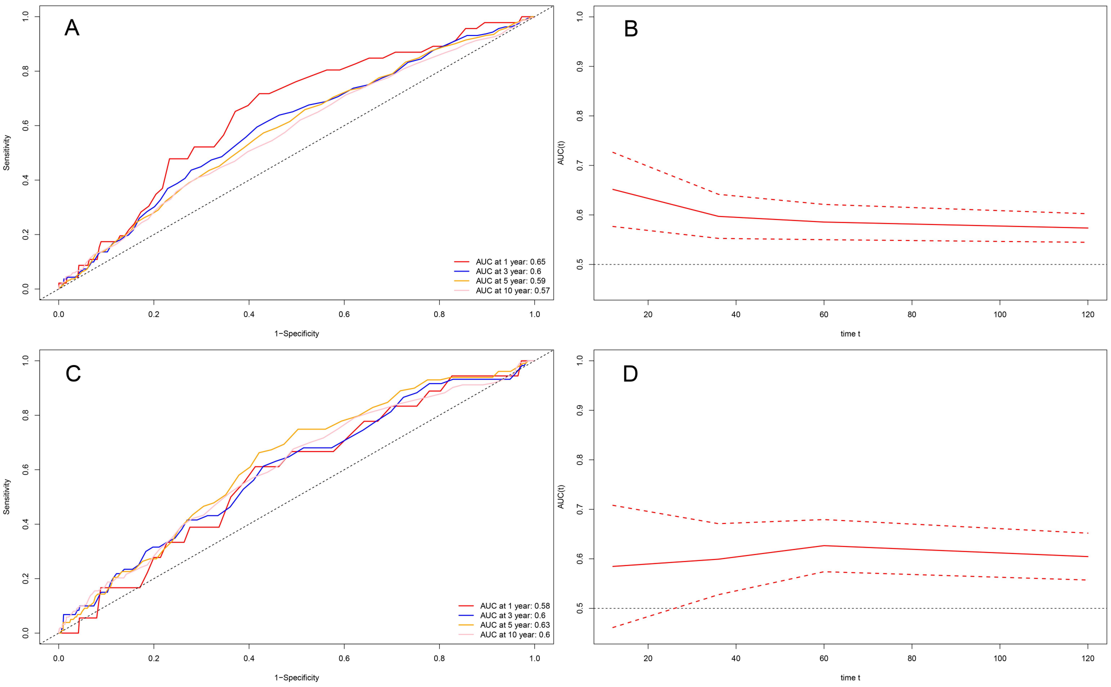

Supplementary Figure S4. Time-dependent receiver operating characteristic curve analysis of neutrophils for mortality prediction in osteoarthritis patients. (A, B) All-cause mortality; (C, D) Cardiovascular mortality.

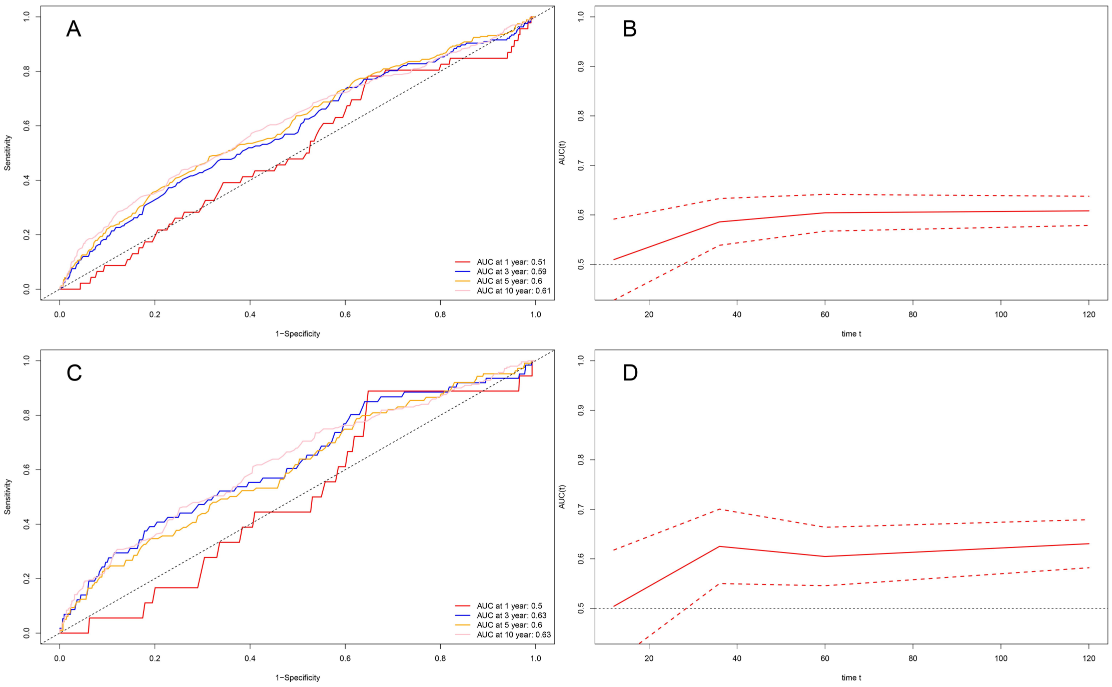

Supplementary Figure S5. Time-dependent receiver operating characteristic curve analysis of platelets for mortality prediction in osteoarthritis patients. (A, B) All-cause mortality; (C, D) Cardiovascular mortality.

Supplementary Tables

Supplementary Table S1. Weighted Cox regression analysis of the relationship between the neutrophil-lymphocyte ratio and mortality in osteoarthritis patients after excluding patients who died within 2 years.

	NLR levels				
	Lower NLR (< 2.53)	Higher NLR (≥ 2.53)	P value	Per 1 increment	P value
All-cause mortality					
Model 1	1 (ref)	1.68 (1.38, 2.05)	<0.001	1.15 (1.08, 1.23)	<0.001
Model 2	1 (ref)	1.66 (1.37, 2.02)	<0.001	1.14 (1.07, 1.22)	<0.001
Cardiovascular mortality					
Model 1	1 (ref)	2.39 (1.62, 3.53)	<0.001	1.22 (1.12, 1.33)	<0.001
Model 2	1 (ref)	2.40 (1.68, 3.44)	<0.001	1.22 (1.11, 1.35)	<0.001

Model 1: adjusted for age and sex. Model 2: adjusted for age, sex, race, education level, smoking status, drinking status, BMI, diabetes, and hypertension data. NLR: neutrophil-lymphocyte ratio; BMI: body mass index.

Supplementary Table S2. Weighted Cox regression analysis of the relationship between the neutrophil-lymphocyte ratio and mortality in osteoarthritis patients after excluding patients with diabetes.

	NLR levels			Per 1 increment	P value
	Lower NLR (< 2.53)	Higher NLR (≥ 2.53)	P value		
All-cause mortality					
Model 1	1 (ref)	1.76 (1.43, 2.17)	<0.001	1.14 (1.07, 1.21)	<0.001
Model 2	1 (ref)	1.75 (1.41, 2.18)	<0.001	1.14 (1.07, 1.22)	<0.001
Cardiovascular mortality					
Model 1	1 (ref)	2.51 (1.62, 3.90)	<0.001	1.16 (1.07, 1.25)	<0.001
Model 2	1 (ref)	2.47 (1.55, 3.93)	<0.001	1.17 (1.08, 1.28)	<0.001

Model 1: adjusted for age and sex data. Model 2: adjusted for age, sex, race, education level, smoking status, drinking status, BMI, and hypertension data. NLR: neutrophil-lymphocyte ratio; BMI: body mass index.
